# Vitamin D, Thyroid Hormones and Cardiovascular Risk: Exploring the Components of This Novel Disease Triangle

**DOI:** 10.3389/fphys.2021.722912

**Published:** 2021-09-16

**Authors:** Cristina Vassalle, Alessandra Parlanti, Alessandro Pingitore, Sergio Berti, Giorgio Iervasi, Laura Sabatino

**Affiliations:** ^1^Fondazione CNR-Regione Toscana Gabriele Monasterio, Pisa, Italy; ^2^Institute of Clinical Physiology, CNR, Pisa, Italy

**Keywords:** vitamin D, 25(OH)D, thyroid hormones, cardiovascular system, pathophysiology

## Abstract

The role of thyroid hormones (THs) in the cardiovascular (CV) system, through several direct and indirect effects is recognized. Even very small modification in TH levels (as those observed in subclinical hypothyroidism or hyperthyroidism, and low triiodothyronine syndrome) may adversely affect the CV system, whereas thyroid hormones benefit the CV system and improve the prognosis. There is also evidence of vitamin D effects on cardiometabolic disease (*e.g.*, through modulation of endothelial and smooth muscle cell activity, renin-angiotensin-aldosterone system, nitric oxide, oxidative stress, and inflammatory response), as well as an association between vitamin D [25(OH)D] deficiency and autoimmune thyroid diseases or cancer, and a relationship between vitamin D concentration and titers of antibodies and thyroid autoimmunity replacement. Interestingly, experimental data indicate a direct effect of vitamin D on Type 2 deiodinase expression causing subsequential peripheral conversion of T4 into T3. However, the functional links among THs, vitamin D and the cardiovascular system, and clinical effects of coexisting abnormalities in this new troublesome triad, have not yet been reviewed. The main aim of this review is to discuss pathophysiology of this relationship, proposing new mechanistic insights involving vitamin D in the modulation of cardiometabolic disease and thyroid profile.

## Introduction

The roles of thyroid hormones (THs) in cardiovascular (CV) disease, such as heart failure (HF) or acute myocardial infarction (MI), through several direct and indirect effects are well-known (Jabbar et al., 2017; Abdel-Moneim et al., 2020). The two conditions share a number of underlying mechanisms and risk factors (*e.g.*, endothelial dysfunction, increased blood pressure and dyslipidemia) (Jabbar et al., 2017). Moreover, the importance of THs in CV homeostasis may be deduced by the fact that even very small changes in TH levels (*e.g.*, those observed in subclinical hypothyroidism or hyperthyroidism, and low triiodothyronine syndrome) adversely impact the CV system, whereas THs benefit the CV system and improve the prognosis (Razvi et al., 2018; Mastorci et al., 2020). Moreover, whether experimental studies suggest that TH administration may reduce infarct size and improve myocardial function after acute myocardial infarction (AMI), increasing clinical evidence which indicates that the manifestations of subtle thyroid abnormalities (*e.g.*, low T3 syndrome) during AMI course are associated with adverse prognosis (Razvi et al., 2018).

In this context, the classical roles of vitamin D are related to the regulation of bone turnover and phospho-calcium homeostasis (Caprio et al., 2017). However, the pleiotropic roles of vitamin D have been recognized through preclinical and observational studies, and the importance of vitamin D in non-skeletal sites has emerged, as well as the role of vitamin D in type 2 diabetes (T2D) and cardiovascular and autoimmune diseases, neoplasia, and all-cause mortality (Caprio et al., 2017). Many data indicate that Vitamin D affects cardiometabolic disease. Its actions include modulation of endothelial and smooth muscle cell activity, renin-angiotensin-aldosterone system (RAAS), insulin, nitric oxide, oxidative stress status, inflammatory response, as well as aortic valve calcification processes (Kim et al., 2017; Latic and Erben, 2020; de la Guía-Galipienso et al., 2020). Its deficiency has been associated with CV risk, a higher risk of atherosclerosis, HF and CV mortality (Latic and Erben, 2020; de la Guía-Galipienso et al., 2020).

Moreover, there are some evidence suggesting an association between vitamin D [25(OH)D, the form by which vitamin D status is measured in blood] deficiency and autoimmune thyroid diseases (*e.g.*, Hashimoto’s thyroiditis and Graves’ disease, and/or postpartum thyroiditis), and a relationship between vitamin D levels and titers of antibodies and thyroid autoimmunity replacement (Kim, 2017). Experimental results suggest that vitamin D status could exert a role on thyroid cancer onset and progression, and that the active form of vitamin D [1,25(OH)2D3] might be beneficial in thyroid cancer treatment (Kim, 2017). Interestingly, vitamin D has been shown to be associated with an increase of Type 2 deiodinase (DIO2) levels and peripheral conversion of THs (specifically from thyroxine-T4 into triiodothyronine-T3) in tissue homogenates (from liver, kidney, muscle, femur bone, heart and brain) of diabetic rats (Alrefaie and Awad, 2015). Moreover, although all the mechanisms underlying the role of vitamin D on TH profile are not completely understood, likely there may be the involvement of vitamin D-related antioxidants, anti-inflammatory and immunoregulatory effects (Kim, 2017).

The functional links among THs, vitamin D and the cardiovascular system, and clinical effects of coexisting abnormalities in this new troublesome triad, have not yet been reviewed. The main aim of this review is to discuss pathophysiology of this triangle, proposing new mechanistic insights involving vitamin D in the onset and development of cardiometabolic disease and TH function.

## THs and CV System

TH system has a central regulatory role on virtually all metabolic functions in the human body by interacting with several cellular pathways, TH facilitate the functional integration among different organs and systems, in various physiological and pathological conditions, involving autonomic nervous system, the renin-angiotensin-aldosterone system, vascular reactivity and renal function (Razvi et al., 2018). At CV level, TH regulate homeostasis mainly by influencing cardiac contractility and systemic vascular resistance. In chronic diseases, at systemic level, TH promote regenerative and reparative processes to compensate the systemic stress conditions (Sabatino et al., 2014). In this perspective, TH play as multiple level regulators and, thus, have a relevant potential for innovative therapeutic approaches (Pantos et al., 2007b, 2010). T3 is considered the biologically active form of TH and mediates almost all TH effects at tissue level. T3 is generated by 5′-monodeiodination of T4 in peripheral tissues by type I (DIO1) and DIO2 deiodinases. A third enzyme, called type III 5-monodeiodinase (DIO3) is responsible of TH catabolism and catalyzes the removal of one iodine from the inner tyrosilic ring of T4 and its conversion to the inactive rT3 (reverse-T3) (Bianco and da Conceicao, 2018). THs homeostasis in the heart requires a fine regulation, involving specific transporters at plasma membranes (such as the monocarboxylate transporters MCT8 and MCT10) (Friesema et al., 2008). The finding of monodeiodinases activities in normal and pathological myocytes unveiled a more complex TH regulatory dynamic than initially thought, since it suggested that both hormones (not only the active T3) can be uploaded inside the cardiac cells by membrane transporters (Sabatino et al., 2000; Gereben et al., 2008). THs effects at genomic level are mediated by specific receptors (TRs): TRα (TRα1, TRα2), TRβ (TRβ1, TRβ2): TH-TR complex specifically interacts with TH-response elements (TREs) in the promoters of target genes (Cheng et al., 2010). Differently, the non-genomic action of TH trigger specific metabolic pathways involving receptors at plasma membrane level (*i.e.*, integrin ανβ3) or cytosolic TRs (Pantos and Mourouzis, 2014). Recently, the protective effects of THs have been investigated in rats where the alteration of molecular function and biological processes turned out to be beneficial after MI (Pantos et al., 2007a). THs has been shown to increase cardiac contractility, stimulate cardiac hypertrophy and angiogenesis, reduce apoptosis, improve left ventricular remodeling and function. More specifically, T3 regulates myocardial contraction/relaxation in MI by inducing the pathological switch of myosin heavy chains (MHC) from α to β isoform, and controls calcium ions (Ca^2+^) flux into the sarcoplasmic reticulum by inducing expression of sarcoplasmic reticulum calcium adenosine triphosphatase (SERCA2) and inhibiting phospholamban (PLB), SERCA’s counteracting molecule (Kinugawa et al., 2001). Interestingly, the overexpression of TRα1 during the pathological hypertrophy settlement in MI in rats is strictly associated to this receptor redistribution from nucleus to cytoplasm, where it plays an important role in the (MHC) from α to β isoform switch (Kinugawa et al., 2001).

Besides the effects on the heart, TH genomic and, mainly, non-genomic effects are also evident on the vasculature, both in vascular smooth muscle (VSMC) and endothelial cells (Ojamaa et al., 1996; Sabatino et al., 2015). The rapidity with which VSMC respond to T3 suggest the involvement of main non-genomic mechanisms, since the genomic would require a longer period for protein synthesis and for biological effects to be observed (Ojamaa et al., 1996). It is well known that T3 induces vascular relaxation via endothelium-dependent pathways. However, more recently, several studies have demonstrated that T3 may potentiate vascular relaxation also through endothelium-independent mechanisms, in which VSMC are a crucial target for T3-mediated vasodilation (Samuel et al., 2017). More specifically, nitric oxide (NO) production by VSMC, other than by endothelium, has been considered the driving factor in local regulation of vascular response (Lee et al., 2008) mainly via PI3K/Akt signaling pathway activation (Carrillo-Sepúlveda et al., 2010). Furthermore, it has been observed that both T4 and T3 administration induce new blood vessel formation via surface receptor ανβ3-mediated pathway in human microvascular endothelial cells (Balzan et al., 2013; Liu et al., 2014) and in rat heart and aorta tissues during ischemic reperfusion procedure (Sabatino et al., 2016).

## Vitamin D and CV Pathophysiology

Vitamin D receptors are present in a variety of cells and tissues, including cardiomyocytes, VSMC and endothelial cells, suggesting many extra-skeletal effects of this vitamin, over the traditional calcium and phosphorus homeostasis (Gouni-Berthold and Berthold, 2021). Indeed, several data reported a correlation between vitamin D deficiency and CV disease, as 25(OH)D levels appeared associated with different CV risk factors (e.g., blood pressure, obesity, and metabolic syndrome), incidence of atherosclerosis and heart failure, peripheral arterial disease, and adverse prognosis and mortality, suggesting that the indirect association between hypovitaminosis D with the cardiovascular system is essentially explained through its association with cardiometabolic risk factors (Vassalle et al., 2015). In particular, the effects of vitamin D on diabetes include improvement of insulin sensitivity (*e.g.*, through modulation of adiponectin gene), increase of insulin gene transcription, insulin receptor expression, and glucose transport (Maestro et al., 2000; Lahbib et al., 2015; Lontchi-Yimagou et al., 2020). In humans, 25(OH)D inversely correlates with glycated hemoglobin, and with prevalence and risk of diabetes (Zhao et al., 2020; Bejar et al., 2021; Salih et al., 2021).

**FIGURE 1:**
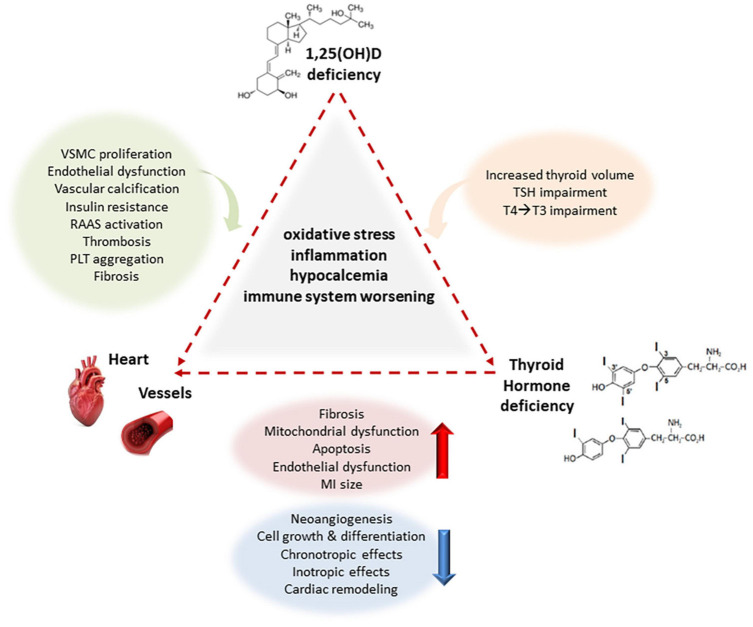
Main interactions between deficiency of vitamin D and THs on the CV system creating a complex network in a adverse vicious circle. Under the effect of underlying common mechanisms related to oxidative stress, inflammation, hypocalcemia and immune system worsening, Vitamin D deficiency induces VSMC proliferation, endothelial dysfunction, facilitates vascular calcification, and promotes insulin resistance, RAAS activation, thrombosis, PLT aggregation, and fibrosis to the CV system, and increased thyroid volume, TSH and T4/T3 impairment to thyroid, whereas THs deficiency also induces fibrosis, mitochondrial dysfunction, apoptosis, endothelial dysfunction, reduces chronotropic and inotropic effects, increasing MI size in experimental models, and reducing eNOS expression and activity, angiogenesis, cellular growth and differentiation, and cardiac remodeling at cardiovascular level. VSMC vascular smooth muscle cells, eNOS endothelial nitric oxide synthase, RAAS renin–angiotensin–aldosterone system, MI myocardial infarction, TSH thyroid stimulating hormone, T3 triiodothyronine, T4 thyroxine.

Over the course of time, it has been estimated that calcitriol directly or indirectly regulates a myriad of genes, modulating a number of pathophysiological pathways. Both genomic and non-genomic actions are mediated by the vitamin D receptor (VDR), through regulation of transcriptional activity of target genes or activation of intracellular second messengers (Pike et al., 2016). Nonetheless, the modulation of gene expression may be regulated by co-regulatory elements rather than only by VDR (Pike et al., 2016). Thus, it is difficult to list all possible effects mediated by vitamin D on the CV system. In fact, many possible mechanisms may explain effects of vitamin D on CV systems through effects on endothelium (e.g., vitamin D deficiency causes reduced eNOS expression and SOD activity), smooth muscle cells and vascular calcification (e.g., modulating metalloproteinase expression), RAAS activation, effects on altered inflammatory pathways (e.g., vitamin D deficiency is associate with increased of NF-kB and IL-6), insulin secretion and sensitivity (e.g., reducing inflammation and oxidative stress, and regulates Ca^2+^ level in different cellular types as well as adipokines release such as adiponectin and leptin), parathyroid hormone (PTH) secretion [1,25(OH)2D regulates its own synthesis decreasing the PTH synthesis and secretion] (Jablonski et al., 2011; Aoshima et al., 2012; Szeto et al., 2012; Vassalle and Pérez-López, 2013; Wacker and Holiack, 2013; Lim et al., 2020; Szymczak-Pajor et al., 2020; Wee et al., 2021) (Figure 1).

Chronic vitamin D deficiency may predispose to hypertension, and one mechanism recently identified characterizing this direct cardiac effects is the modulation of the function of transient receptor potential C cation channels, which is a mechanosensitive cation channel that plays a role in cardiac slow-force responses to hemodynamic changes (Stratford et al., 2021). Moreover, vitamin D has direct effects on the atrial electrophysiology and atrial fibrillation, a direct association with regulation of cardiac autonomic activity, and a direct role in the regulation of vasomotor inducing changes of pressor and depressor vasomotor responses (Mann et al., 2013; Hanafy et al., 2014). Some studies evidenced that vitamin D deficiency results in structural and ionic channel remodeling and autonomic dysfunction that may predispose the individuals to lethal cardiac arrhythmias and sudden cardiac death (Drechsler et al., 2010; Deo et al., 2011; Mann et al., 2013). In fact, vitamin D has direct effects on myocytes. Vitamin D also affects cardiac contractility indirectly by calcium metabolism and directly via vitamin D receptors-VDR (Tishkoff et al., 2008).

For it concerns atherosclerosis, vitamin D can act in several ways. For example, macrophages lacking VDR present an increase in the uptake of cholesterol and this results in cholesterol accumulating in the intracellular space, whereas knockdown of VDR in endothelial cells leads to endothelial cell activation (upregulation of VCAM-1, ICAM-1 and IL-6, decreased peripheral blood mononuclear cell-PBMC rolling velocity and increased PBMC rolling flux and adhesion to the endothelial line) (Szeto et al., 2012; Bozic et al., 2015).

Interestingly, among direct effects, recent data suggest that vitamin D maintains endothelial stability *in vitro* by enforcing cell-cell interactions (Vila Cuenca et al., 2018).

However, although several studies have demonstrated beneficial effects of vitamin D supplementation (versus placebo) on CV clinical manifestation and risk factors, no clear benefit of vitamin D administration for CV disease has been evidenced in randomized controlled trials (Latic and Erben, 2020; Michos et al., 2021).

Surely further work is needed to establish the protective role of vitamin D in this setting, as controversial results may be due to interference of possible confounding factors, such as baseline 25(OH)D levels, dose, duration of supplementation, season, as well as population characteristics (*e.g.*, number of subjects enrolled, heterogeneity of patients, time spent outdoors), which may be considered in result interpretation.

## Vitamin D and Thyroid Pathophysiology

The finding relative to homology between the vitamin D receptor (VDR) and the TH receptor dates back to the late 1980s (McDonnell et al., 1988). Few years later, the VDR expression on rat follicular thyroid cells was evidenced, suggesting a possible vitamin D role in thyroid pathophysiology (Berg et al., 1994). However, until today, most data that evaluated the role of vitamin D in thyroid illnesses are only in the context of autoimmunity and cancer (recently reviewed in Kim et al., 2017; Mele et al., 2020).

### Autoimmune Thyroid Disease (AITD)

Hypovitaminosis D appears involved in the pathogenesis of autoimmune disease, where the most common autoimmune thyroid diseases are Hashimoto’s thyroiditis (HT) and Grave’s disease (GD).

Hashimoto’s thyroiditis (HT) is characterized by lymphocytic infiltration destroying the gland tissue, inflammation, and release of thyroid antibodies (TPOAb and TgAb), leading to hypothyroidism.

A meta-analysis [including 6/3 studies with continuous/dichotomous 25(OH)D levels] showed that HT patients had lower values compared to controls, and more likely have 25(OH)D deficiency (Wang et al., 2015). Moreover, data on the inverse correlation between vitamin D values with thyroid volume, and duration and severity of HT, and TH and thyroid antibody levels further reinforcing the idea that vitamin D deficiency might play a role in the risk, onset and development of HT (Muscogiuri et al., 2016; Chao et al., 2020).

Interestingly, several polymorphisms in the VDR gene (*e.g.*, the most common *Bsm*I, *Fok*I, *Taq*I, and *Apa*I) have been studied in their association with HT risk (O’Grady et al., 2014; Giovinazzo et al., 2017; Wang et al., 2017). Although the results are controversial and far from definitive, these data suggested the genetically determined low vitamin D might be associated with an increased risk of developing HT.

All together these data suggested the possibility of the use of vitamin D supplementation against HT. Much still needs to be done in this context, because available data retain many pitfalls, as the study populations are generally small, patients heterogeneous, there is variability of administration (*e.g.*, dose and timing of supplementation), and many confounding factors may affect final results (*e.g.*, age, gender, obesity, lifestyle, baseline vitamin D status, univocity of categories to define hypovitaminosis, season) are often neglected. Moreover, if values higher than 30 ng/mL (75 nmol/L) are considered optimal for bone and mineral homeostasis health, correct levels to lower the risk of autoimmune disease onset and progression are largely unknown (Holick et al., 2011). However, to testify to this possibility, reduction of TPOAb and TgAb levels after cholecalciferol supplementation was observed in patients with low vitamin D (Simsek et al., 2016; Krysiak et al., 2018; Wang et al., 2018).

Grave’s disease (GD) is characterized by the presence of gland infiltration by T lymphocytes, thyroid stimulating hormone (TSH) receptor autoantibodies (TRAb), goiter, and ophthalmopathy, and hyperthyroidism.

Two meta-analyses suggested that a low vitamin D status may increase the risk of GD (Wang et al., 2015; Xu et al., 2015). Moreover, there is data on the association between low vitamin D levels and the thyroid autoantibody titre in patients with Graves’ disease as well as the association of polymorphisms in the vitamin D receptor (VDR) gene in patients with GD (Unal et al., 2014; Zhang et al., 2015; Veneti et al., 2019; Płazińska et al., 2020).

For it concerns vitamin D supplementation, there are a few studies in the GD setting. In one study, GD patients treated with standard therapy combined with calcitriol [methimazole 30 mg/d supplemented with 1.5 micrograms α(OH)D3 for 24 weeks] showed a greater decrease in serum FT4 and FT3, although there were no differences in TRAb levels compared to those taking only methimazole (Kawakami-Tani et al., 1997). Interestingly, a recent study suggested that administration of cholecalciferol (1000–2000 IU/day) in GD patients with hypovitaminosis D, if not decrease the recurrence rate within one year, determines an earlier occurrence in the group of GD patients not taking cholecalciferol (Cho and Chung, 2020).

### Thyroid Cancer

Vitamin D seems to play a critical role in thyroid cancer onset and progression, acting with effects on a number of cellular pathways, including apoptosis, cellular proliferation and differentiation, angiogenesis, oxidative stress and inflammatory response (Mele et al., 2020). However, despite the strong evidence observed in experimental models, clinical studies give more uncertain results (Mele et al., 2020). Nonetheless, two recent meta-analyses evidenced the association between low vitamin D levels and increased risk for thyroid cancer (Hu et al., 2018; Zhao et al., 2019). Instead, more unclear are the studies investigating the role of vitamin D supplementation in preventing thyroid cancer onset, which remain inconclusive (Zhang et al., 2013; O’Grady et al., 2014).

### Central and Peripheral Effects on Thyroid Function

Little is known on the relationship between vitamin D to other thyroid dysfunction or disease.

It has been observed that Vitamin D may affect FRTL-5 cells (thyroid *cell* line established from normal thyroid glands in rats) inhibiting TSH-stimulated adenylyl cyclase activity, iodide uptake and cell growth (Berg et al., 1994). Moreover, vitamin D3 administration decreased TSH values toward normal in diabetic rats (52 ± 13, 70 ± 19, 57 ± 19 μIU/ml in control group, diabetic group and diabetic group treated with vitamin D3, respectively) (Alrefaie and Awad, 2015). Accordingly, an inverse correlation between 25(OH)D levels and TSH was observed in general human populations (Chailurkit et al., 2013; Barchetta et al., 2015).

A central effect of 1,25(OH)2 vitamin D3 has been observed in the receptor modulation (VDR) of TSH secretion by rat pituitary thyrotroph cells (Sar et al., 1980; Smith et al., 1989). In this context, experimental data suggested that vitamin D could increase TRH-induced TSH secretion by pituitary thyrotroph cells (D’Emden and Wark, 1987). Despite these effects, it is also possible that the inverse low TSH in the presence of a high vitamin D could be due, almost in part, to increased THs caused by the stimulatory effect of vitamin D on thyrocytes, and the consequent negative feedback control that the THs exert over the hypothalamus and anterior pituitary, thus controlling the release of both thyrotropin releasing hormone (TRH) and TSH (Dietrich et al., 2012; Veneti et al., 2019).

Interestingly, vitamin D administration improved TH profile in diabetic rats, increasing DIO2 (Alrefaie and Awad, 2015). This effect of vitamin treatment was also observed in bone extracts from mice skeleton, where DIO2 activity appeared increased by 2 to 3-fold, and in primary osteoblastic cells, where 1,25(OH)2D3 dose- and time-dependently induced the mRNA expression of DIO2 (Miura et al., 2002; Gouveia et al., 2005).

**FIGURE 2:**
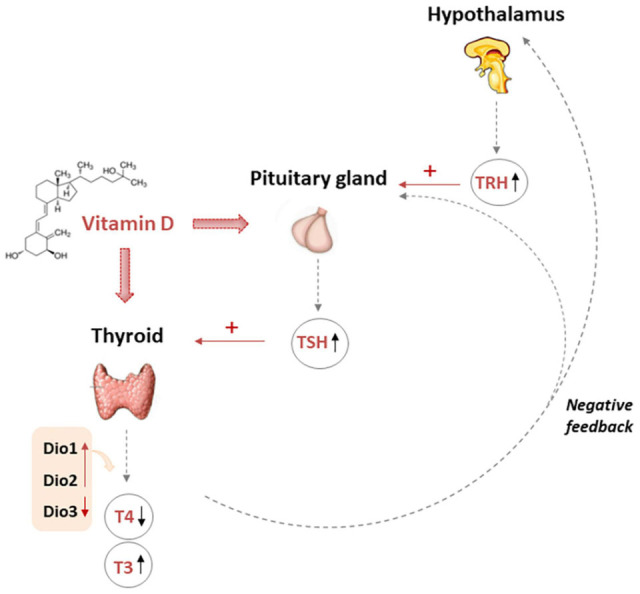
Multiple action of vitamin D affecting TH levels. The pituitary gland secretes TSH that stimulates the thyroid to produce thyroxine (T4) and triiodothyronine (T3). Moreover T3 may be produced in peripheral organs by deiodination from circulating T4. Vitamin D fits in the complex mechanisms of regulation of THs. In fact, vitamin D could increase TRH-induced-TSH secretion by pituitary thyrotroph cells. However, it is also possible that the finding of low TSH in the presence of a high vitamin D could be due, almost in part, to increased THs caused by the stimulatory effect of vitamin D on thyrocytes, and the activation of the negative feedback control that the THs exert over the hypothalamus and pituitary gland, thus finely modulating the release of TRH, TSH, and the TH themselves. TRH Thyrotropin-releasing hormone, TSH thyroid stimulating hormone, T3 triiodothyronine, T4 thyroxine, DIO deiodinase.

All together these data suggested how vitamin D may be important, acting at central level for pituitary gland function, also modulating DIO2 expression at thyroid and other organ levels and consequently affecting peripheral conversion of T4 into T3 (Figure 2).

However, if is true that vitamin D may affects thyroid pathophysiology, this relationship is likely reciprocal, although less is understood on the influence of THs on vitamin D metabolism. It is known that excess of THs stimulates bone resorption, increasing the blood values of calcium and phosphorus and suppressing PTH secretion whereas in hypothyroidism the bone turnover is decreased, the serum calcium concentration tends to be lower, and PTH secretion activated (Delitala et al., 2020; Hans and Levine, 2021). Accordingly, some data suggested that THs can affect vitamin D metabolism and, consequently, modulate the availability of its active form 1,25-(OH)2D3 (decreased in the serum of hyperthyroid patients and increased in the serum of untreated hypothyroid patients), which appeared normalized upon restoration of normal thyroid function (Bouillon et al., 1980).

## Vitamin D in the Relationship Between Thyroid and CV System

In GD patients, a significant reduction of pulse wave velocity (PWV, index of arterial stiffness, recognized as a CV risk factor) was observed among vitamin D insufficient participants after cholecalciferol supplementation (70 μg/day-2800 IU for 9 months; Grove-Laugesen et al., 2019). Other results suggested a role for hypovitaminosis D through actions on visceral fat and insulin resistance in the interaction between THs and metabolic syndrome, with consequently increase of cardiometabolic risk (Verrusio et al., 2019). Moreover, an interaction between vitamin D, insulin resistance and thyroid profile dysfunction was observed in obese subjects, likely driven by systemic inflammation, where vitamin D deficiency represented the only independent factor associated with presence of HT in such patients (Răcătăianu et al., 2018). Another study, conducted in a general population, suggested that different combinations of vitamin D and TH status can modulate changes in the lipid profile (triglycerides and HDL) (Mansorian et al., 2018). Clearly, the verification and identification of these correlations as causality relations is required, with the design of interventional or cohort studies to provide benchmark evidence.

In this context, the case of Low T3 Syndrome (LT3) after AMI is interesting for its clinical significance (Jabbar et al., 2017). LT3 syndrome is characterized by an isolated reduction of blood T3 concentration with normal T4 and TSH levels (Jabbar et al., 2017). This condition interests about 20% of acute MI patients, and it is associated with more severe clinical manifestations (left ventricular dysfunction, large MI, and inflammatory and stress responses), and adverse prognosis (rate of major cardiac events and a short- and long-term mortality) (Razvi et al., 2018). Changes in blood THs after acute MI may be the result of increased DIO3 activity and reduced DIO1 and DIO2 activities (Olivares et al., 2007; Van den Berghe, 2014). At present, the utility of TH therapy in the management of acute MI is evaluated in early-phase clinical trials for improvement of TH profile, although also looking after possible important adverse effects related to supraphysiological doses as adverse effects in the application of this treatment (Razvi et al., 2018). Thus, in this context, the possibility to use vitamin D supplementation (safe and very rarely toxic even at high doses) instead or in combination with TH treatment in acute MI settings may be of interest. We evidenced that there is a relationship between LT3 syndrome and hypovitaminosis D in acute MI patients (submitted data). In the same population, we observed that patients with low vitamin D (deficient and insufficient groups) showed a trend toward higher TSH levels in comparison to patients who had adequate or optimal levels of vitamin D (1.8 ± 1.8 *versus* 1.4 ± 1.0), finding particularly evident in those with a severe hypovitaminosis D (< 10ng/L), although without reaching significance values (likely the number of patients enrolled, *n* = 120, needs to be increased) (Table 1). Moreover, patients had higher TSH levels in the Spring-Winter period as opposed to the Autumn-Summer season, according to previous data consistently reporting a significant TSH increase in the colder periods (Table 1; Maes et al., 1997; Das et al., 2018). Higher TSH levels in colder seasons can be due, almost partially, to TSH hypersecretion in response to lower ambient temperature, lower peripheral metabolism and change in diet and physical activity (Danforth et al., 1979; Shinomiya et al., 2014; Louzada et al., 2014). However, we also observed an inverse seasonal trend for 25(OH)D and TSH, as previously reported (Table 1; Barchetta et al., 2015; Das et al., 2018). This fact may suggest that vitamin D may have a role in the modulation of TSH secretion, similarly to its regulatory role on other pituitary hormones (*e.g.*, growth hormone; Esposito et al., 2019).

**TABLE 1:** TSH values according to 25(OH)D status in the overall population and in different seasons.

		**<10**	**10–30**	**>30 ng/L**
	All patients	1.83 ± 1.6	1.78 ± 2.0	1.42 ± 1.0
**TSH (mU/L)**				
mean ± DS	Winter-Spring	1.93 ± 1.9	1.92 ± 2.2	1.44 ± 1.1
	Summer-Autumn	1.7 ± 1.2	1.3 ± 0.5	1.3 ± 0.7

**FIGURE 3:**
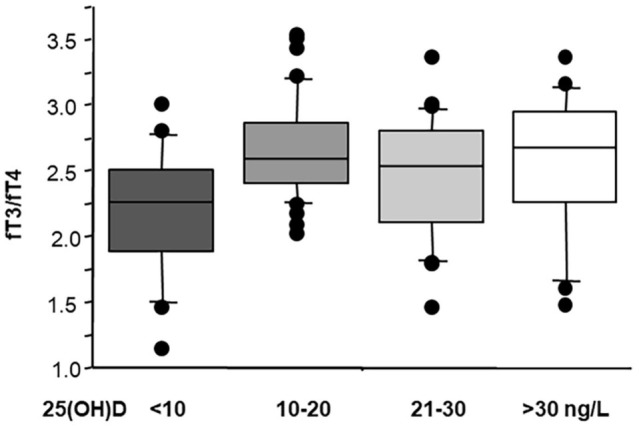
Values of fT3/fT4 according to 25(OH)D status in 124 AMI patients (ANOVA; severe hypovitaminosis D versus normal vitamin D *p* ≤ 0.001). Median, interquartile, outliers, and extremes of fT3/fT4 are given.

Notably, patients with a severe hypovitaminosis D [25(OH)D < 10 ng/L] showed lowest fT3/fT4 levels (a TH index, which reflect deiodinase activity and conversion of T4 to T3), suggesting that this category of patients may require more attention and could benefit more from 25(OH)D supplementation (Figure 3). This fact is further confirmed by previous data, showing that stable coronary artery disease prevalence and severity (multivessel disease) was particularly higher in patients with severe hypovitaminosis D (<10 ng/ml) (Verdoia et al., 2014, 2021; Nardin et al., 2016), where the effects obtained through restoration of sufficient 25(OH)D levels may be further evaluated in future studies.

Moreover, we also observed that acute MI patients in the group of severe hypovitaminosis D are older than the subjects belonging to the other categories (ANOVA; *p* < 0.01) and more frequently present T2D (43%, ANOVA; *p* < 0.01). Interestingly, patients in the group of 25(OH)D > 30ng/L do not present T2D, suggesting a negative predictive value for T2D in those with sufficient vitamin D status in this population.

## Vitamin D and Infectious and Other Immune-Related Diseases

A relationship between 25-hydroxyvitamin D hypovitaminosis and increased risk of developing different immune-related conditions and infectious diseases has been observed, and recently reviewed (Charoenngam and Holick, 2020). In particular low 25(OH)D levels have been associated to psoriasis, type 1 diabetes, multiple sclerosis, rheumatoid arthritis, tuberculosis, sepsis, respiratory infection, and also COVID-19 (Ponsonby et al., 2005; Megna et al., 2020; Cristelo et al., 2021; Jaimni et al., 2021; Wald et al., 2021; Chetty and Chetty, 2021).

From these observations, many attempts have been made to utilize vitamin D supplementation as a treatment or co-treatment in addition to traditional immunomodulatory drugs. Unfortunately, there are not yet shared protocols od 25(OH)D supplementation in the different conditions, also due to the many variables that take place [*e.g*., dose, route of administration, dietary interference, genetic individual characteristics, and age, body mass index, season and sunlight exposure, baseline levels of 25(OH)D, patient typology, and supplementation time, and type of outcome measurements]. Thus, available data are heterogeneous, and as such not definitive and sometimes controversial (Charoenngam and Holick, 2020).

Moreover, for it concerns association between vitamin D and Sars-CoV-2 infection, emerging data indicate that severe vitamin D deficiency resulted more common in COVID-19 patients, playing a significant role in worsening the prognosis of these patients, suggesting vitamin D supplementation as a possible prophylactic and therapy in this setting (Grant et al., 2020; Brito et al., 2021; Campi et al., 2021; Güven and Gültekin, 2021). On this regard, a previous meta-analysis evidenced that vitamin D supplementation resulted safe and protective against acute respiratory tract infection, with patients with severely deficiency experienced the major benefit (Martineau et al., 2017). For COVID-19 patients, a dosage of 50,000 IU of vitamin D3 twice a week for the first week and the dose of 50,000 IU for the second and third week was suggested to reach and maintain levels above 40 ng/ml (Ebadi and Montano-Loza, 2020). Interestingly, a small cohort observational study evidenced that combined oral doses of vitamin D (1000 IU), Mg (150 mg), and vitamin B12 (500 μg) was associated with a significant reduction in the proportion of COVID patients with clinical worsening (requiring oxygen support, intensive care support, or both) (Tan et al., 2020).

Interestingly, seen the adverse repercussions of Sars-CoV-2 infection on the cardiovascular system and thyroid function, whether the use of supplementation to maintain sufficient vitamin D levels may alleviate these conditions and their complications in COVID-19 patients remains to be better elucidated (Gorini et al., 2020; Kumari et al., 2020; Zhang et al., 2020; Ansari Ramandi et al., 2021).

## Conclusion

For many years medicine has been developing into separate, specialized, almost independent branches. However, much evidence reveals significant connections between organs, until now considered as single entities, revealing a complex intermingled network with a common list of causes, or contributing factors for “different diseases,” attesting for shared cellular and molecular mechanisms for their etiology and progression. So, we must approach a more holistic view to better understand pathophysiology and improve care of our patients. Certainly, a lot of aspects are not clear. Currently, there is not enough strong evidence to support screening for vitamin D deficiency or vitamin D supplementation in thyroid profile and CV disease setting with the purpose of risk reduction, or improvement of these conditions. Several factors will be still considered in future studies, such as seasonal adjustment of vitamin D levels, and variable populations and definite reference 25(OH)D values for deficiency in extra-skeletal conditions, such as CV disease. Moreover, most studies are based on a single measurement of serum vitamin D levels, which may not be the best indicator of 25(OH)D over time. Nonetheless, even considering the deepening of these aspects, available evidence suggested a role of vitamin D in the relationship between THs and cardiometabolic disease, providing vitamin D as a potential additional, effective, low-cost, easily available and safe tool to be evaluate in the clinical practice for multi-organ and multi-disease benefits.

## Methods

We have performed a search for articles in PubMed to identify pertinent literature, till 13 August 2021, with the following primary keywords: “COVID-19,” “Sars-CoV-2,” “vitamin D,” “25(OH)D,” “thyroid,” “cardiovascular disease,” “supplementation,” “thyroid hormones,” with interposition of the Boolean operator “AND.”

## Author Contributions

CV conceived the manuscript and wrote the original draft. LS wrote the TH/CV part and took care of the graphical figure development. All authors read and approved the final version of the manuscript.

## Conflict of Interest

The authors declare that the research was conducted in the absence of any commercial or financial relationships that could be construed as a potential conflict of interest.

## Publisher’s Note

All claims expressed in this article are solely those of the authors and do not necessarily represent those of their affiliated organizations, or those of the publisher, the editors and the reviewers. Any product that may be evaluated in this article, or claim that may be made by its manufacturer, is not guaranteed or endorsed by the publisher.

## References

[B1] Abdel-Moneim A. Gaber A. M. Gouda S. Osama A. Othman S. I. Allam G. (2020). Relationship of thyroid dysfunction with cardiovascular diseases: updated review on heart failure progression. *Hormones* 19 301–309. 10.1007/s42000-020-00208-8 32488814

[B2] Alrefaie Z. Awad H. (2015). Effect of vitamin D3 on thyroid function and deiodinase 2 expression in diabetic rats. *Arch. Physiol. Biochem.* 121 206–209. 10.3109/13813455.2015.1107101 26599099

[B3] Ansari Ramandi M. M. Yarmohammadi H. Beikmohammadi S. Hosseiny Fahimi B. H. Hatami F. Beydokhti H. (2021). Comparison of the cardiovascular presentations, complications and outcomes following different coronaviruses’ infection: A systematic review. *J. Cardiovasc. Thorac. Res.* 3 92–101. 10.34172/jcvtr.2021.29 34326962 PMC8302895

[B4] Aoshima Y. Mizobuchi M. Ogata H. Kumata C. Nakazawa A. Kondo F. (2012). Vitamin D receptor activators inhibit vascular smooth muscle cell mineralization induced by phosphate and TNF-α. *Nephrol. Dial. Transplant.* 27 1800–1806. 10.1093/ndt/gfr758 22287655

[B5] Balzan S. Del Carratore R. Nardulli C. Sabatino L. Lubrano V. Iervasi G. (2013). The Stimulative Effect of T3 and T4 on Human Myocardial Endothelial Cell Proliferation, Migration and Angiogenesis. *J. Clin. Exp. Cardiolog.* 4:12.

[B6] Barchetta I. Baroni M. G. Leonetti F. De Bernardinis M. Bertoccini L. Fontana M. (2015). TSH levels are associated with vitamin D status and seasonality in an adult population of euthyroid adults. *Clin. Exp. Med.* 15 389–396. 10.1007/s10238-014-0290-9 24925636

[B7] Bejar C. A. Goyal S. Afzal S. Mangino M. Zhou A. van der Most P. J. (2021). A Bidirectional Mendelian Randomization Study to evaluate the causal role of reduced blood vitamin D levels with type 2 diabetes risk in South Asians and Europeans. *Nutr. J.* 20:71. 34315477 10.1186/s12937-021-00725-1 PMC8314596

[B8] Berg J. P. Liane K. M. Bjørhovde S. B. Bjøro T. Torjesen P. A. Haug E. (1994). Vitamin D receptor binding and biological effects of cholecalciferol analogues in rat thyroid cells. *J. Steroid. Biochem. Mol. Biol.* 50 145–150. 10.1016/0960-0760(94)90021-3 8049143

[B9] Bianco A. C. da Conceicao R. R. (2018). The deiodinase trio and thyroid hormone signaling. *Methods. Mol. Biol.* 1810 67–83. 10.1007/978-1-4939-7902-88 PMC6668716 29892818

[B10] Bouillon R. Muls E. De Moor P. (1980). Influence of thyroid function on the serum concentration of 1,25-dihydroxyvitamin D3. *J. Clin. Endocrinol. Metab.* 51 793–797. 10.1210/jcem-51-4-793 6893457

[B11] Bozic M. Álvarez Á de Pablo C. Sanchez-Niño M. D. Ortiz A. Dolcet X. (2015). Impaired Vitamin D Signaling in Endothelial Cell Leads to an Enhanced Leukocyte-Endothelium Interplay: Implications for Atherosclerosis Development. *PLoS One* 10:e0136863. 10.1371/journal.pone.0136863 26322890 PMC4556440

[B12] Brito D. T. M. Ribeiro L. H. C. Daltro C. H. D. C. Silva R. B. (2021). The possible benefits of vitamin D in COVID-19. *Nutrition.* 91-92:111356. 10.1016/j.nut.2021.111356 34352586 PMC8149468

[B13] Campi I. Gennari L. Merlotti D. Mingiano C. Frosali A. Giovanelli L. (2021). Vitamin D and COVID-19 severity and related mortality: a prospective study in Italy. *BMC Infect. Dis.* 21:566. 34126960 10.1186/s12879-021-06281-7 PMC8200788

[B14] Caprio M. Infante M. Calanchini M. Mammi C. Fabbri A. (2017). Vitamin D: not just the bone. Evidence for beneficial pleiotropic extraskeletal effects. *Eat. Weight. Disord.* 22 27–41. 10.1007/s40519-016-0312-6 27553017

[B15] Carrillo-Sepúlveda M. A. Ceravolo G. S. Fortes Z. B. Carvalho M. H. Tostes R. C. Laurindo F. R. (2010). Thyroid hormone stimulates NO production via activation of the PI3K/Akt pathway in vascular myocytes. *Cardiovasc. Res.* 85 560–570. 10.1093/cvr/cvp304 19734167 PMC3031311

[B16] Chailurkit L. O. Aekplakorn W. Ongphiphadhanakul B. (2013). High vitamin D status in younger individuals is associated with low circulating thyrotropin. *Thyroid* 23 25–30. 10.1089/thy.2012.0001 22931506

[B17] Chao G. Zhu Y. Fang L. (2020). Correlation Between Hashimoto’s Thyroiditis-Related Thyroid Hormone Levels and 25-Hydroxyvitamin D. *Front. Endocrinol.* 11:4. 10.3389/fendo.2020.00004 PMC7034299 32117049

[B18] Charoenngam N. Holick M. F. (2020). Immunologic Effects of Vitamin D on Human Health and Disease. *Nutrients* 12:2097. 10.3390/nu12072097 32679784 PMC7400911

[B19] Cheng S. Y. Leonard J. L. Davis P. J. (2010). Molecular aspects of thyroid hormone actions. *Endocr. Rev.* 31 139–170. 10.1210/er.2009-0007 20051527 PMC2852208

[B20] Chetty V. V. Chetty M. (2021). Potential benefit of vitamin d supplementation in people with respiratory illnesses, during the Covid-19 pandemic. *Clin. Transl. Sci.* 31:13044. 10.1111/cts.13044 34057814 PMC8239894

[B21] Cho Y. Y. Chung Y. J. (2020). Vitamin D supplementation does not prevent the recurrence of Graves’ disease. *Sci. Rep.* 10:16. 31913301 10.1038/s41598-019-55107-9 PMC6949266

[B22] Cristelo C. Machado A. Sarmento B. Gama F. M. (2021). The roles of vitamin D and cathelicidin in type 1 diabetes susceptibility. *Endocr. Connect.* 10 R1–R12. 33263562 10.1530/EC-20-0484 PMC7923048

[B23] Danforth E. Horton E. S. O’ Connell M. Vagenakis A. G. (1979). Dietary induced alterations in thyroid hormone metabolism during over nutrition. *J. Clin. Invest.* 64 1336–1347. 10.1172/jci109590 500814 PMC371281

[B24] Das G. Taylor P. N. Javaid H. Tennant B. P. Geen J. Aldridge A. (2018). Seasonal variation of vitamin d and serum thyrotropin levels and its relationship in a euthyroid caucasian population. *Endocr Pract.* 24 53–59. 10.4158/ep-2017-0058 29144817

[B25] de la Guía-Galipienso F. Martínez-Ferran M. Vallecillo N. Lavie C. J. Sanchis-Gomar F. Pareja-Galeano H. (2020). Vitamin D and cardiovascular health. *Clin Nutr.* 20 30700–30707. 10.1016/j.clnu.2020.12.025 PMC7770490 33397599

[B26] Delitala A. P. Scuteri A. Doria C. (2020). Thyroid Hormone Diseases and Osteoporosis. *J. Clin. Med.* 9:1034. 10.3390/jcm9041034 32268542 PMC7230461

[B27] D’Emden M. C. Wark J. D. (1987). 1,25-Dihydroxyvitamin D3 enhances thyrotropin releasing hormone induced thyrotropin secretion in normal pituitary cells. *Endocrinology* 121 1192–1194. 10.1210/endo-121-3-1192 3113918

[B28] Deo R. Katz R. Shlipak M. G. Sotoodehnia N. Psaty B. M. Sarnak M. J. (2011). Vitamin D parathyroid hormone, and sudden cardiac death: results from the Cardiovascular Health Study. *Hypertension* 58 1021–1028. 10.1161/hypertensionaha.111.179135 22068871 PMC3337033

[B29] Dietrich J. W. Landgrafe G. Fotiadou E. H. (2012). TSH and Thyrotropic Agonists: Key Actors in Thyroid Homeostasis. *J. Thyroid. Res.* 2012:351864. 23365787 10.1155/2012/351864 PMC3544290

[B30] Drechsler C. Pilz S. Obermayer-Pietsch B. Verduijn M. Tomaschitz A. Krane V. (2010). Vitamin D deficiency is associated with sudden cardiac death, combined cardiovascular events, and mortality in haemodialysis patients. *Eur. Heart J.* 31 2253–2261. 10.1093/eurheartj/ehq246 20688781 PMC2938469

[B31] Ebadi M. Montano-Loza A. J. (2020). Perspective: improving vitamin D status in the management of COVID-19. *Eur. J. Clin. Nutr.* 74 856–859. 10.1038/s41430-020-0661-0 32398871 PMC7216123

[B32] Esposito S. Leonardi A. Lanciotti L. Cofini M. Muzi G. Penta L. (2019). Vitamin D and growth hormone in children: a review of the current scientific knowledge. *J. Transl. Med.* 17:87. 10.1159/000060832 30885216 PMC6421660

[B33] Friesema E. C. H. Jansen J. Jachtenberg J. W. Visser W. E. Kester M. H. A. Visser T. J. (2008). Effective Cellular Uptake and Efflux of Thyroid Hormone by Human Monocarboxylate Transporter 10. *Mol. Endocrinol.* 22 1357–1369. 10.1210/me.2007-0112 18337592 PMC5419535

[B34] Gereben B. Zavacki A. M. Ribich S. Kim B. W. Huang S. A. Simonides W. S. (2008). Cellular and molecular basis of deiodinase-regulated thyroid hormone signaling. *Endocr. Rev.* 29 898–938. 10.1210/er.2008-0019 18815314 PMC2647704

[B35] Giovinazzo S. Vicchio T. M. Certo R. Alibrandi A. Palmieri O. Campennì A. (2017). Vitamin D receptor gene polymorphisms/haplotypes and serum 25(OH)D3 levels in Hashimoto’s thyroiditis. *Endocrine* 55 599–606. 10.1007/s12020-016-0942-5 27043843

[B36] Gorini F. Bianchi F. Iervasi G. (2020). COVID-19 and Thyroid: Progress and Prospects. *Int. J. Environ. Res. Public. Health.* 17:6630. 10.3390/ijerph17186630 32932954 PMC7559547

[B37] Gouni-Berthold I. Berthold H. K. (2021). Vitamin D and Vascular Disease. *Curr. Vasc. Pharmacol.* 19 250–268. 10.2174/1570161118666200317151955 32183681

[B38] Gouveia C. H. Christoffolete M. A. Zaitune C. R. Dora J. M. Harney J. W. Maia A. L. (2005). Type 2 iodothyronine selenodeiodinase is expressed throughout the mouse skeleton and in the MC3T3-E1 mouse osteoblastic cell line during differentiation. *Endocrinology* 146 195–200. 10.1210/en.2004-1043 15471965

[B39] Grant W. B. Lahore H. McDonnellm S. L. Baggerly C. A. French C. B. Aliano J. L. (2020). Evidence that Vitamin D Supplementation Could Reduce Risk of Influenza and COVID-19 Infections and Deaths. *Nutrients* 12:988. 10.3390/nu12040988 32492787 PMC7352449

[B40] Grove-Laugesen D. Malmstroem S. Ebbehoj E. Riis A. L. Watt T. Hansen K. W. (2019). Effect of 9 months of vitamin D supplementation on arterial stiffness and blood pressure in Graves’ disease: a randomized clinical trial. *Endocrine* 66 386–397. 10.1007/s12020-019-01997-8 31280470

[B41] Güven M. Gültekin H. (2021). Association of 25-Hydroxyvitamin D Level with COVID-19-Related in-Hospital Mortality: A Retrospective Cohort Study. *J. Am. Coll. Nutr.* 9 1–10. 10.1080/07315724.2021.1935361 34370620

[B42] Hanafy D. A. Chang S. L. Lu Y. Y. Chen Y. C. Kao Y. H. Huang J. H. (2014). Electromechanical effects of 1,25-dihydroxyvitamin d with antiatrial fibrillation activities. *J. Cardiovasc. Electrophysiol.* 25 317–323. 10.1111/jce.12309 24152033

[B43] Hans S. K. Levine S. N. (2021). *Hypoparathyroidism.* Treasure Island (FL): StatPearls Publishing. 28722928

[B44] Holick M. F. Binkley N. C. Bischoff-Ferrari H. A. Gordon C. M. Hanley D. A. Heaney R. P. (2011). Evaluation, treatment, and prevention of vitamin D deficiency: an Endocrine Society clinical practice guideline. *J. Clin. Endocrinol. Metab.* 96 1911–1930. 10.1210/jc.2011-0385 21646368

[B45] Hu M. J. Zhang Q. Liang L. Wang S. Y. Zheng X. C. Zhou M. M. (2018). Association between vitamin D deficiency and risk of thyroid cancer: a case-control study and a meta-analysis. *J. Endocrinol. Invest.* 41 1199–1210. 10.1007/s40618-018-0853-9 29464660

[B46] Jabbar A. Pingitore A. Pearce S. H. Zaman A. Iervasi G. Razvi S. (2017). Thyroid hormones and cardiovascular disease. *Nat. Rev. Cardiol.* 14 39–55. 27811932 10.1038/nrcardio.2016.174

[B47] Jablonski K. L. Chonchol M. Pierce G. L. Walker A. E. Seals D. R. (2011). 25-Hydroxyvitamin D deficiency is associated with inflammation-linked vascular endothelial dysfunction in middle-aged and older adults. *Hypertension* 57 63–69. 10.1161/hypertensionaha.110.160929 21115878 PMC3020150

[B48] Jaimni V. Shasty B. A. Madhyastha S. P. Shetty G. V. Acharya R. V. Bekur R. (2021). Association of Vitamin D Deficiency and Newly Diagnosed Pulmonary Tuberculosis. *Pulm. Med.* 2021:5285841. 33510909 10.1155/2021/5285841 PMC7826226

[B49] Kawakami-Tani T. Fukawa E. Tanaka H. Abe Y. Makino I. (1997). Effect of 1 alpha-hydroxyvitamin D3 on serum levels of thyroid hormones in hyperthyroid patients with untreated Graves’ disease. *Metabolism* 46 1184–1188. 10.1016/s0026-0495(97)90214-6 9322804

[B50] Kim D. (2017). The Role of Vitamin D in Thyroid Diseases. *Int. J. Mol. Sci.* 18:1949. 10.3390/ijms18091949 28895880 PMC5618598

[B51] Kim J. K. Park M. J. Song Y. R. Kim H. J. Kim S. G. (2017). Vitamin D: a possible modifying factor linking obesity to vascular calcification in hemodialysis patients. *Nutr. Metab.* 14:27. 10.1186/s12986-017-0181-7 PMC5356240 28331532

[B52] Kinugawa K. Yonekura K. Ribeiro R. C. Eto Y. Aoyagi T. Baxter J. D. (2001). Regulation of thyroid hormone receptor isoforms in physiological and pathological cardiac hypertrophy. *Circ. Res.* 89 591–598. 10.1161/hh1901.096706 11577024

[B53] Krysiak R. Kowalcze K. Okopień B. (2018). Selenomethionine potentiates the impact of vitamin D on thyroid autoimmunity in euthyroid women with Hashimoto’s thyroiditis and low vitamin D status. *Pharmacol. Rep.* 71 367–373. 10.1016/j.pharep.2018.12.006 30844687

[B54] Kumari K. Chainy G. B. N. Subudhi U. (2020). Prospective role of thyroid disorders in monitoring COVID-19 pandemic. *Heliyon* 6:e05712. 10.1016/j.heliyon.2020.e05712 33344794 PMC7733548

[B55] Lahbib A. Ghodbane S. Louchami K. Sener A. Sakly M. Abdelmelek H. (2015). Effects of vitamin D on insulin secretion and glucose transporter GLUT2 under static magnetic field in rat. *Environ. Sci. Pollut. Res. Int.* 22 18011–18016. 10.1007/s11356-015-4844-5 26169817

[B56] Latic N. Erben R. G. (2020). Vitamin D and Cardiovascular Disease, with Emphasis on Hypertension, Atherosclerosis, and Heart Failure. *Int. J. Mol. Sci.* 21:6483. 10.3390/ijms21186483 32899880 PMC7555466

[B57] Lee J. H. Xia S. Ragolia L. (2008). Upregulation of AT2 receptor and iNOS impairs angiotensin II-induced contraction without endothelium influence in young normotensive diabetic rats. *Am. J. Physiol.* 295 R144–R154. 10.1152/ajpregu.00191.2008 PMC2494818 18463192

[B58] Lim K. Molostvov G. Lubczanska M. Fletcher S. Bland R. Hiemstra T. F. (2020). Impaired arterial vitamin D signaling occurs in the development of vascular calcification. *PLoS One* 15:e0241976. 10.1371/journal.pone.0241976 33211721 PMC7676703

[B59] Liu X. Zheng N. Shi Y. N. Yuan J. Li L. (2014). Thyroid hormone induced angiogenesis through the integrin αvβ3/protein kinase D/histone deacetylase 5 signaling pathway. *J. Mol. Endocrinol.* 52 245–254. 10.1530/jme-13-0252 24532656

[B60] Lontchi-Yimagou E. Kang S. Goyal A. Zhang K. You J. Y. Carey M. (2020). Insulin-sensitizing effects of vitamin D repletion mediated by adipocyte vitamin D receptor: Studies in humans and mice. *Mol. Metab.* 42:101095. 10.1016/j.molmet.2020.101095 33045433 PMC7585951

[B61] Louzada R. A. Santos M. C. Cavalcanti de Albuquerque J. P. Rangel I. F. Ferreira A. C. Galina A. (2014). Type 2 iodothyronine deiodinase is up regulated in rat slow and fast twitch skeletal muscle during cold exposure. *Am. J. Physiol. Endocrinol. Metab.* 307 E1020–E1029. 25294216 10.1152/ajpendo.00637.2013

[B62] Maes M. Mommen K. Hendrickx D. Peeters D. D’Hondt P. Ranjan R. (1997). Components of biological variation, including seasonality, in blood concentrations of TSH. FT3, FT4, PRL, cortisol and testosterone in healthy volunteers. *Clin. Endocrinol.* 46 587–598. 10.1046/j.1365-2265.1997.1881002.x 9231055

[B63] Maestro B. Campión J. Dávila N. Calle C. (2000). Stimulation by 1,25-dihydroxyvitamin D3 of insulin receptor expression and insulin responsiveness for glucose transport in U-937 human promonocytic cells. *Endocr. J.* 47 383–391. 10.1507/endocrj.47.383 11075718

[B64] Mann M. C. Exner D. V. Hemmelgarn B. R. Sola D. Y. Turin T. C. Ellis L. (2013). Vitamin D levels are associated with cardiac autonomic activity in healty humans. *Nutrients* 5 2114–2127. 10.3390/nu5062114 23752493 PMC3725496

[B65] Mansorian B. Mirza-Aghazadeh Attari M. Vahabzadeh D. Mohebbi I. (2018). Serum vitamin D level and its relation to thyroid hormone, blood sugar and lipid profiles in Iranian sedentary work staff. *Nutr. Hosp.* 35 1107–1114. 10.20960/nh.1719 30307294

[B66] Martineau A. R. Jolliffe D. A. Hooper R. L. Greenberg L. Aloia J. F. Bergman P. (2017). Vitamin D supplementation to prevent acute respiratory tract infections: systematic review and meta-analysis of individual participant data. *BMJ* 356:i6583. 10.1136/bmj.i6583 28202713 PMC5310969

[B67] Mastorci F. Sabatino L. Vassalle C. Pingitore A. (2020). Cardioprotection and Thyroid Hormones in the Clinical Setting of Heart Failure. *Front. Endocrinol.* 10:927. 10.3389/fendo.2019.00927 PMC6997485 32047475

[B68] McDonnell D. P. Pike J. W. O’Malley B. W. (1988). The vitamin D receptor: A primitive steroid receptor related to thyroid hormone receptor. *J. Steroid. Biochem.* 30 41–46. 10.1016/0022-4731(88)90074-x 2838696

[B69] Megna M. Ferrillo M. Barrea L. Patruno C. Muscogiuri G. Savastano S. (2020). Vitamin D and psoriasis: an update for dermatologists and nutritionists. *Minerva. Endocrinol.* 45 138–147. 32340428 10.23736/S0391-1977.20.03190-9

[B70] Mele C. Caputo M. Bisceglia A. Samà M. T. Zavattaro M. Aimaretti G. (2020). Immunomodulatory Effects of Vitamin D in Thyroid Diseases. *Nutrients* 12:1444. 10.3390/nu12051444 32429416 PMC7284826

[B71] Michos E. D. Cainzos-Achirica M. Heravi A. S. Appel L. J. (2021). Vitamin D, Calcium Supplements, and Implications for Cardiovascular Health: JACC Focus Seminar. *J. Am. Coll. Cardiol.* 77 437–449. 33509400 10.1016/j.jacc.2020.09.617

[B72] Miura M. Tanaka K. Komatsu Y. Suda M. Yasoda A. Sakuma Y. (2002). A novel interaction between thyroid hormones and 1,25(OH)(2)D(3) in osteoclast formation. *Biochem. Biophys. Res. Commun.* 291 987–994. 10.1006/bbrc.2002.6561 11866463

[B73] Muscogiuri G. Mari D. Prolo S. Fatti L. M. Cantone M. C. Garagnani P. (2016). 25 Hydroxyvitamin D Deficiency and Its Relationship to Autoimmune Thyroid Disease in the Elderly. *Int. J. Environ. Res. Public Health* 13:850. 10.3390/ijerph13090850 27571093 PMC5036683

[B74] Nardin M. Verdoia M. Schaffer A. Barbieri L. Marino P. De Luca G. (2016). Vitamin D status, diabetes mellitus and coronary artery disease in patients undergoing coronary angiography. *Atherosclerosis* 250 114–121. 10.1016/j.atherosclerosis.2016.05.019 27205868

[B75] O’Grady T. J. Kitahara C. M. DiRienzo A. G. Gates M. A. (2014). The association between selenium and other micronutrients and thyroid cancer incidence in the NIH-AARP Diet and Health Study. *PLoS One* 9:e110886. 10.1371/journal.pone.0110886 25329812 PMC4203851

[B76] Ojamaa K. Klemperer J. D. Klein I. (1996). Acute Effects of Thyroid Hormone on Vascular Smooth Muscle. *Thyroid* 6 505–512. 10.1089/thy.1996.6.505 8936680

[B77] Olivares E. L. Marassi M. P. Fortunato R. S. da Silva A. C. Costa-e-Sousa R. H. Araújo I. G. (2007). Thyroid function disturbance and type 3 iodothyronine deiodinase induction after myocardial infarction in rats a time course study. *Endocrinology* 148 4786–4792. 10.1210/en.2007-0043 17628010

[B78] Pantos C. Mourouzis I. (2014). The emerging role of TRα1 in cardiac repair: potential therapeutic implications. *Oxid. Med. Cell. Longev.* 2014:481482. 10.1155/2014/481482 24683435 PMC3941156

[B79] Pantos C. Mourouzis I. Cokkinos D. V. (2010). Thyroid hormone as a therapeutic option for treating ischaemic heart disease: from early reperfusion to late remodelling. *Vascul. Pharmacol.* 52 157–165. 10.1016/j.vph.2009.11.006 19951746

[B80] Pantos C. Mourouzis I. Markakis K. Dimopoulos A. Xinaris C. Kokkinos A. D. (2007a). Thyroid hormone attenuates cardiac remodeling and improves hemodynamics early after acute myocardial infarction in rats. *Eur. J. Cardiothorac. Surg.* 32 333–339. 10.1016/j.ejcts.2007.05.004 17560116

[B81] Pantos C. Xinaris C. Mourouzis I. Malliopoulou V. Kardami E. Cokkinos D. V. (2007b). Thyroid hormone changes cardiomyocyte shape and geometry via ERK signaling pathway: Potential therapeutic implications in reversing cardiac remodeling? *Mol. Cell. Biochem.* 297 65–72. 10.1007/s11010-006-9323-3 17024559

[B82] Pike J. W. Meyer M. B. Benkusky N. A. Lee S. M. St John H. Carlson A. (2016). Genomic Determinants of Vitamin D-Regulated Gene Expression. *Vitam. Horm.* 100 21–44. 10.1016/bs.vh.2015.10.011 26827947 PMC5113140

[B83] Płazińska M. T. Czarnywojtek A. Sawicka-Gutaj N. Zgorzalewicz-Stachowiak M. Czarnocka B. Gut P. (2020). Vitamin D deficiency and thyroid autoantibody fluctuations in patients with Graves’ disease - A mere coincidence or a real relationship? *Adv. Med. Sci.* 65 39–45. 10.1016/j.advms.2019.11.001 31884304

[B84] Ponsonby A. L. Lucas R. M. van der Mei I. A. (2005). UVR, vitamin D and three autoimmune diseases–multiple sclerosis, type 1 diabetes, rheumatoid arthritis. *Photochem. Photobiol.* 81 1267–1275. 10.1562/2005-02-15-ir-441 15971932

[B85] Răcătăianu N. Leach N. V. Bolboacă S. D. Cozma A. Dronca E. Valea A. (2018). Vitamin D deficiency, insulin resistance and thyroid dysfunction in obese patients: is inflammation the common link? *Scand. J. Clin. Lab. Invest.* 78 560–565. 10.1080/00365513.2018.1517420 30362842

[B86] Razvi S. Jabbar A. Pingitore A. Danzi S. Biondi B. Klein I. (2018). Thyroid Hormones and Cardiovascular Function and Diseases. *J. Am. Coll. Cardiol.* 71 1781–1796. 29673469 10.1016/j.jacc.2018.02.045

[B87] Sabatino L. Iervasi G. Ferrazzi P. Francesconi D. Chopra I. J. (2000). A study of iodothyronine 5′-monodeiodinase activities in normal and pathological tissues in man and their comparison with activities in rat tissues. *Life. Sci.* 68 191–202. 10.1016/S0024-3205(00)00929-2 11191637

[B88] Sabatino L. Iervasi G. Pingitore A. (2014). Thyroid hormone and heart failure: from myocardial protection to systemic regulation. *Expert. Rev. Cardiovasc. Ther.* 12 1227–1236. 10.1586/14779072.2014.957674 25220579

[B89] Sabatino L. Kusmic C. Nicolini G. Amato R. Casini G. Iervasi G. (2016). T3 enhances Ang2 in rat aorta in myocardial I/R: comparison with left ventricle. *J. Mol. Endocrinol.* 57 139–149. 10.1530/JME-16-0118 27444191

[B90] Sabatino L. Lubrano V. Balzan S. Kusmic C. Del Turco S. Iervasi G. (2015). Thyroid hormone deiodinases D1, D2, and D3 are expressed in human endothelial dermal microvascular line: effects of thyroid hormones. *Mol. Cell. Biochem.* 399 87–94. 10.1007/s11010-014-2235-8 25304215

[B91] Salih Y. A. Rasool M. T. Ahmed I. H. Mohammed A. A. (2021). Impact of vitamin D level on glycemic control in diabetes mellitus type 2 in Duhok. *Ann. Med. Surg.* 64:102208. 10.1016/j.amsu.2021.102208 33786167 PMC7988274

[B92] Samuel S. Zhang K. Tang Y. D. Gerdes A. M. Carrillo-Sepulveda M. A. (2017). Triiodothyronine Potentiates Vasorelaxation via PKG/VASP Signaling in Vascular Smooth Muscle Cells. *Cell. Physiol. Biochem.* 41 1894–1904. 10.1159/000471938 28376489

[B93] Sar M. Stumpf W. E. DeLuca H. F. (1980). Thyrotropes in the pituitary are target cells for 1,25 dihydroxy vitamin D3. *Cell. Tissue Res.* 209 161–166. 7000361 10.1007/BF00219932

[B94] Shinomiya A. Shinmura T. Nishiwaki-Ohkawa T. Yoshimura T. (2014). Regulation of seasonal reproduction by hypothalamic activation of thyroid hormone. *Front. Endocrinol.* 5:12. 10.3389/fendo.2014.00012 PMC3930870 24600435

[B95] Simsek Y. Cakır I. Yetmis M. Dizdar O. S. Baspinar O. Gokay F. (2016). Effects of Vitamin D treatment on thyroid autoimmunity. *J. Res. Med. Sci.* 21:85. 10.4103/1735-1995.192501 28163731 PMC5244647

[B96] Smith M. A. McHenry C. Oslapas R. Hofmann C. Hessel P. Paloyan E. (1989). Altered TSH levels associated with increased serum 1,25-dihydroxyvitamin D3: a possible link between thyroid and parathyroid disease. *Surgery* 106 987–991. 2511635

[B97] Stratford K. Haykal-Coates N. Thompson L. Farraj A. Hazari M. (2021). Early-life persistent vitamin D deficiency-induced cardiovascular dysfunction in mice is mediated by transient receptor potential C channels. *J. Steroid. Biochem. Mol. Biol.* 206:105804. 10.1016/j.jsbmb.2020.105804 33338589 PMC9152789

[B98] Szeto F. L. Reardon C. A. Yoon D. Wang Y. Wong K. E. Chen Y. (2012). Vitamin D receptor signaling inhibits atherosclerosis in mice. *Mol. Endocrinol.* 26 1091–1101. 10.1210/me.2011-1329 22638071 PMC3385794

[B99] Szymczak-Pajor I. Drzewoski J. Śliwińska A. (2020). The Molecular Mechanisms by Which Vitamin D Prevents Insulin Resistance and Associated Disorders. *Int. J. Mol. Sci.* 21:6644. 10.3390/ijms21186644 32932777 PMC7554927

[B100] Tan C. W. Ho L. P. Kalimuddin S. Cherng B. P. Z. Teh Y. E. Thien S. Y. (2020). Cohort study to evaluate the effect of vitamin D, magnesium, and vitamin B12 in combination on progression to severe outcomes in older patients with coronavirus (COVID-19). *Nutrition* 79-80:111017. 10.1016/j.nut.2020.111017 33039952 PMC7832811

[B101] Tishkoff D. X. Nibbelink K. A. Holmberg K. H. Dandu L. Simpson R. U. (2008). Functional vitamin D receptor (VDR) in the t-tubules of cardiac myocytes: VDR knockout cardiomyocyte contractility. *Endocrinology* 149 558–564. 10.1210/en.2007-0805 17974622 PMC2219302

[B102] Unal A. D. Tarcin O. Parildar H. Cigerli O. Eroglu H. Demirag N. G. (2014). Vitamin D deficiency is related to thyroid antibodies in autoimmune thyroiditis. *Cent. Eur. J. Immunol.* 39 493–497. 10.5114/ceji.2014.47735 26155169 PMC4439962

[B103] Van den Berghe G. (2014). Non-thyroidal illness in the ICU: a syndrome with different faces. *Thyroid.* 24 1456–1465. 10.1089/thy.2014.0201 24845024 PMC4195234

[B104] Vassalle C. Maffei S. Iervasi G. (2015). “Bone remodelling biomarkers: new actors on the old cardiovascular stage,” in *The Biomarker Validation – Technological, Clinical and Commercial Aspect*, eds Seitz H. Schumacher S. (Weinheim: Wiley-VCH Verlag GmbH & Co. KGaA), 107–146. 10.1002/9783527680658.ch7

[B105] Vassalle C. Pérez-López F. R. (2013). “The Importance of Some Analytical Aspects and Confounding Factors in Relation to Clinical Interpretation of Results,” in *The Vitamin D: Daily Requirements, Dietary Sources and Symptoms of Deficiency*, eds Meer C. Smits H. (New York, NY:.Nova Publisher), 816–818.

[B106] Veneti S. Anagnostis P. Adamidou F. Artzouchaltzi A. M. Boboridis K. Kita M. (2019). Association between vitamin D receptor gene polymorphisms and Graves’ disease: a systematic review and meta-analysis. *Endocrine* 65 244–251. 10.1007/s12020-019-01902-3 30924084

[B107] Verdoia M. Nardin M. Gioscia R. Saghir Afifeh A. M. Viglione F. Negro F. (2021). Association between vitamin D deficiency and serum Homocysteine levels and its relationship with coronary artery disease. *J. Thromb. Thrombolysis* 2021 1–9. 10.1007/s11239-021-02391-w PMC7859464 33538987

[B108] Verdoia M. Schaffer A. Sartori C. Barbieri L. Cassetti E. Marino P. (2014). Vitamin D deficiency is independently associated with the extent of coronary artery disease. *Eur. J. Clin. Invest.* 44 634–642. 10.1111/eci.12281 24829065

[B109] Verrusio W. Magro V. M. Renzi A. Casciaro B. Andreozzi P. Cacciafesta M. (2019). Thyroid hormones, metabolic syndrome and Vitamin D in middle-aged and older euthyroid subjects: a preliminary study. *Aging Clin. Exp. Res.* 31 1337–1341. 10.1007/s40520-018-1071-1 30406357

[B110] Vila Cuenca M. Ferrantelli E. Meinster E. Pouw S. M. Kovačević I. de Menezes R. X. (2018). Vitamin D Attenuates Endothelial Dysfunction in Uremic Rats and Maintains Human Endothelial Stability. *J. Am. Heart Assoc.* 7:e008776. 30371149 10.1161/JAHA.118.008776 PMC6201442

[B111] Wacker M. Holiack M. F. (2013). Vitamin D-effects on skeletal and extraskeletal health and the need for supplementation. *Nutrients* 5 111–148. 10.3390/nu5010111 23306192 PMC3571641

[B112] Wald E. L. Badke C. M. Hintz L. K. Spewak M. Sanchez-Pinto L. N. (2021). Vitamin therapy in sepsis. *Pediatr. Res.* 31 1–9. 10.1038/s41390-021-01673-6 PMC8325544 34333556

[B113] Wang J. Lv S. Chen G. Gao C. He J. Zhong H. (2015). Meta-analysis of the association between vitamin D and autoimmune thyroid disease. *Nutrients* 7 2485–2498. 10.3390/nu7042485 25854833 PMC4425156

[B114] Wang S. Wu Y. Zuo Z. Zhao Y. Wang K. (2018). The effect of vitamin D supplementation on thyroid autoantibody levels in the treatment of autoimmune thyroiditis: a systematic review and a meta-analysis. *Endocrine* 59 499–505. 10.1007/s12020-018-1532-5 29388046

[B115] Wang X. Cheng W. Ma Y. Zhu J. (2017). Vitamin D receptor gene FokI but not TaqI, ApaI, BsmI polymorphism is associated with Hashimoto’s thyroiditis: a meta-analysis. *Sci. Rep.* 7:41540. 28134349 10.1038/srep41540 PMC5278388

[B116] Wee C. L. Mokhtar S. S. Banga Singh K. K. Rasool A. H. G. (2021). Vitamin D deficiency attenuates endothelial function by reducing antioxidant activity and vascular eNOS expression in the rat microcirculation. *Microvasc. Res.* 138:104227. 10.1016/j.mvr.2021.104227 34324883

[B117] Xu M. Y. Cao B. Yin J. Wang D. F. Chen K. L. Lu Q. B. (2015). Vitamin D and Graves’ disease: A meta-analysis update. *Nutrients* 7 3813–3827. 10.3390/nu7053813 26007334 PMC4446781

[B118] Zhang H. Liang L. Xie Z. (2015). Low Vitamin D Status is Associated with Increased Thyrotropin-Receptor Antibody Titer in Graves Disease. *Endocr. Pract.* 21 258–263. 10.4158/ep14191.or 25370319

[B119] Zhang J. McCullough P. A. Tecson K. M. (2020). Vitamin D deficiency in association with endothelial dysfunction: Implications for patients with COVID-19. *Rev. Cardiovasc. Med.* 21 339–344. 10.31083/j.rcm.2020.03.131 33070539

[B120] Zhang L. R. Sawka A. M. Adams L. Hatfield N. Hung R. J. (2013). Vitamin and mineral supplements and thyroid cancer: A systematic review. *Eur. J. Cancer Prev.* 22 158–168. 10.1097/cej.0b013e32835849b0 22926510

[B121] Zhao H. Zhen Y. Wang Z. Qi L. Li Y. Ren L. (2020). The Relationship Between Vitamin D Deficiency and Glycated Hemoglobin Levels in Patients with Type 2 Diabetes Mellitus. *Diabetes Metab. Syndr. Obes.* 13 3899–3907. 10.2147/dmso.s275673 33116736 PMC7585858

[B122] Zhao J. Wang H. Zhang Z. Zhou X. Yao J. Zhang R. (2019). Vitamin D deficiency as a risk factor for thyroid cancer: A meta-analysis of case-control studies. *Nutrition* 57 5–11. 10.1016/j.nut.2018.04.015 30086436

